# To IV or Not to IV: The Science Behind Intravenous Vitamin Therapy

**DOI:** 10.7759/cureus.86527

**Published:** 2025-06-22

**Authors:** Abdulrahman Alangari

**Affiliations:** 1 Department of Medicine, Prince Sultan Military Medical City, Riyadh, SAU; 2 Department of Internal Medicine, Evercare, Riyadh, SAU

**Keywords:** bioavailability, intravenous, nutrient replenishment, oral supplementation, vitamin therapy

## Abstract

Intravenous (IV) vitamin therapy has become an increasingly popular method for delivering essential micronutrients directly into the bloodstream, bypassing the gastrointestinal tract. This method offers enhanced bioavailability, higher therapeutic dosages, and targeted nutrient replenishment, making it particularly effective for individuals with malabsorption issues and chronic illnesses and those seeking optimal health benefits. In contrast, oral supplementation remains a convenient and practical option for routine vitamin and mineral intake. This article provides an overview of the physiological advantages and disadvantages of IV vitamin therapy, its clinical applications, and the scientific evidence supporting its efficacy.

## Introduction and background

Vitamins and minerals play a critical role in maintaining optimal health, supporting a wide range of physiological functions, including immune defense, energy metabolism, hormone regulation, and cellular repair [1,2]. Deficiencies in essential micronutrients can lead to a variety of health issues, ranging from fatigue and weakened immunity to impaired wound healing and chronic disease progression [3]. Traditionally, these nutrients are obtained through a balanced diet or oral supplementation, both of which remain widely used and recommended for most individuals. However, the bioavailability of nutrients consumed orally, meaning the proportion that is absorbed and utilized by the body, can vary significantly due to numerous factors. These include digestive efficiency, the presence of gastrointestinal (GI) disorders, age-related changes in absorption, enzyme deficiencies, interactions with medications, and specific dietary restrictions (e.g., vegetarianism, food allergies) [4-6]. In such cases, the efficacy of oral supplementation may be diminished, and individuals may not experience the intended physiological benefits despite adequate intake.

Intravenous (IV) nutrient therapy has emerged as a promising alternative, especially in clinical scenarios where rapid and reliable nutrient delivery is needed. By bypassing the GI tract and delivering nutrients directly into the bloodstream, IV vitamin therapy ensures higher bioavailability, allowing for immediate absorption and utilization by tissues and cells [4]. This direct route can result in a faster onset of effects, higher therapeutic concentrations in the blood, and improved outcomes for patients who are acutely ill, recovering from surgery, or suffering from chronic malabsorption syndromes. In addition to clinical use, IV vitamin therapy is increasingly being adopted in non-hospital settings, such as wellness clinics, concierge medicine practices, and even mobile IV services [7]. These offer customized "IV drips" with combinations of vitamins, minerals, antioxidants, and amino acids, marketed for purposes ranging from immune boosting and energy enhancement to detoxification, athletic recovery, and aesthetic benefits like skin hydration and anti-aging.

This rising popularity has sparked a wave of interest and debate within both the scientific and public health communities. While the convenience and perceived benefits of IV vitamin therapy are appealing, particularly for individuals seeking rapid nutrient replenishment, higher dosage capabilities, and enhanced therapeutic effects, there remain important questions about its clinical efficacy, safety, cost-effectiveness, and appropriate indications, especially in otherwise healthy individuals [2,8]. As wellness culture continues to blur the lines between medical treatment and consumer self-care, the need for evidence-based evaluation becomes increasingly important. This article aims to explore the primary reasons behind the growing trend of IV vitamin therapy, advantages and disadvantages, and appropriate applications, and the scientific considerations that must be addressed as this therapy continues to expand in both clinical and lifestyle contexts.

## Review

The history and origins of IV therapy

IV therapy has a long and evolving history that has transformed from risky experimentation to an essential part of modern medical practice. The foundation for this technique was laid in the 17th century when English physician William Harvey described the circulation of blood in 1628, marking the first scientific understanding of the cardiovascular system [9]. Shortly after, early researchers attempted rudimentary blood transfusions using animal blood, but these often resulted in severe reactions or death due to a lack of sterility and immunological knowledge. These early failures led to a halt in IV experimentation for nearly two centuries.

The 19th century marked a pivotal moment for IV therapy, particularly during the cholera epidemics of the 1830s [10]. Scottish physician Dr. Thomas Latta developed a method to infuse saline solution directly into the bloodstream to counteract extreme dehydration caused by cholera, effectively saving lives and setting a precedent for fluid resuscitation [11]. Although Latta's work demonstrated the potential of IV therapy, the lack of sterile technique and reliable equipment limited its widespread use. This began to change in the late 19th and early 20th centuries, with the development of hypodermic needles, rubber tubing, and antiseptic methods, all of which helped reduce the risks of infection and enabled safer IV access.

By the early 20th century, IV therapy had become a standard medical practice, especially in surgery, emergency care, and for the treatment of conditions such as dehydration, infections, and nutrient deficiencies. It provided a vital route for delivering blood products, medications, and nutrition when oral intake was not possible. World War I and II significantly accelerated the refinement and adoption of IV techniques, as battlefield medicine necessitated quick and efficient fluid and medication delivery to wounded soldiers [12,13]. The ability to provide rapid, controlled treatment directly into the bloodstream revolutionized acute care medicine. In more recent decades, IV therapy has expanded beyond hospitals and into consumer wellness culture, marketed as a quick fix for fatigue, hangovers, and skin health, despite limited scientific evidence supporting these elective uses.

Why has IV vitamin therapy become fashionable?

Several anecdotal reports suggest that IV vitamin therapy may provide subjective improvements in energy levels, mental clarity, and overall well-being following administration. Users commonly describe faster recovery from conditions such as dehydration, jet lag, hangovers, and physical exhaustion when treated with IV fluids enriched with electrolytes, B vitamins, or vitamin C [7]. Additionally, some individuals report cosmetic benefits, including enhanced skin hydration and a reduction in fatigue, which has contributed to the perception of IV vitamin therapy as a rejuvenating wellness intervention. These claims, although widely publicized by celebrities and influencers, lack support from large-scale, randomized controlled trials and are primarily based on self-reported experiences rather than clinical evidence. Despite the absence of strong empirical validation, the growing popularity of IV vitamin therapy in consumer wellness culture continues to be driven by these anecdotal endorsements and the appeal of rapid, perceived health benefits.

The challenge of oral nutrient absorption

Effective nutrient absorption through the oral route relies on a complex interplay of biochemical and physiological factors. First, GI health significantly affects the body's ability to absorb essential vitamins and minerals. A healthy gastric environment, characterized by sufficient acidity and digestive enzyme activity, is vital for breaking down nutrients into absorbable forms [14]. Conditions such as atrophic gastritis, which reduces stomach acidity, can notably impair the absorption of critical nutrients like vitamin C, iron, and vitamin B12 [15,16]. Conversely, excessive stomach acidity can lead to impaired fat digestion, thus hindering the absorption of fat-soluble vitamins A, D, E, and K. Additionally, conditions like inflammatory bowel diseases (IBD) and celiac disease damage intestinal mucosal integrity, severely affecting the absorption of both macro- and micronutrients. Disorders such as irritable bowel syndrome (IBS) and various dietary intolerances often necessitate dietary restrictions, leading to nutrient deficiencies even in individuals adhering to balanced diets [3].

Another critical factor influencing oral nutrient bioavailability is the liver's first-pass metabolism. After absorption through the intestinal wall, many nutrients, particularly water-soluble vitamins like vitamin C and the B-complex group, undergo extensive metabolism in the liver before entering systemic circulation. This hepatic metabolism significantly reduces their bioavailability, limiting their effective concentration in the bloodstream [14]. Additionally, the absorption of fat-soluble vitamins (A, D, E, K) depends heavily on adequate bile acid production and efficient fat digestion. Individuals with gallbladder disease, liver dysfunction, or related conditions may experience compromised absorption of these essential nutrients [3].

The gut microbiota also exerts considerable influence on nutrient absorption. A balanced gut microbial environment facilitates the breakdown and conversion of nutrients into bioavailable forms. Disturbances such as dysbiosis, leaky gut syndrome, or prolonged antibiotic usage can disrupt this microbial balance, leading to reduced synthesis and impaired absorption of various vitamins [17]. Furthermore, enzyme deficiencies, including lactase deficiency (lactose intolerance) or pancreatic insufficiency, can substantially impair nutrient absorption by decreasing the body's ability to adequately digest and utilize essential dietary components [18].

Finally, the presence of nutrient interactions and competitive absorption pathways can further diminish nutrient uptake. Certain vitamins and minerals share common absorption mechanisms, leading to competitive inhibition. For example, calcium and magnesium compete for the same intestinal transport pathways, so high doses of one mineral may significantly inhibit absorption of the other [6,16,19]. Additionally, while high-fiber diets promote digestive health, dietary fiber can bind to minerals such as zinc, iron, and magnesium, further reducing their availability and absorption [16]. 

Considering these multifaceted challenges, it is not uncommon for individuals to struggle to achieve optimal nutrient status through oral supplementation alone, despite consuming high-quality vitamins and minerals.

IV vitamin therapy

IV nutrient therapy provides an efficient and direct pathway for delivering essential vitamins and minerals into the bloodstream by bypassing the GI tract entirely. This administration route offers several notable advantages over traditional oral supplementation.

Advantages of IV vitamin therapy

A primary benefit of IV vitamin therapy is its superior bioavailability and absorption efficiency. Bioavailability refers to the fraction of nutrients effectively absorbed and utilized by the body. Oral supplements must traverse the GI tract, where their absorption can be significantly affected by factors such as gastric acidity, digestive enzyme activity, the health of the gut microbiota, and underlying medical conditions. For example, conditions such as Crohn's disease, celiac disease, IBS, and other chronic inflammatory or malabsorptive disorders frequently hinder nutrient absorption, drastically reducing the efficacy of oral supplementation [20]. Conversely, nutrients administered intravenously have virtually 100% bioavailability, directly entering systemic circulation without being subjected to digestive processes. This feature is particularly advantageous for patients with GI disorders, post-surgical complications (e.g., bariatric surgery), or chronic illnesses that impair nutrient absorption [14,15,20].

Furthermore, IV nutrient therapy circumvents hepatic first-pass metabolism, a significant limitation of oral supplements. Nutrients given orally must first pass through the liver, where substantial metabolism occurs, reducing their potency and bioavailability. High-dose vitamin C exemplifies this phenomenon. Oral intake exceeding approximately one gram results in significantly diminished absorption due to limited intestinal transporter capacity [3,14]. In contrast, IV-administered vitamin C retains full potency, allowing for much higher therapeutic concentrations in plasma. This direct delivery method ensures rapid cellular uptake, resulting in immediate physiological benefits such as enhanced energy production (associated with B-complex vitamins and magnesium), improved immune function (vitamin C, zinc, and glutathione), reduced oxidative stress and inflammation (glutathione and alphalipoic acid), and improved cognitive function and mental clarity (vitamin B12, NAD+, and magnesium). Consequently, individuals experiencing chronic fatigue, fibromyalgia, or compromised immunity frequently report notable improvements in energy and overall well-being shortly after IV therapy sessions [1,21].

Another significant advantage of IV vitamin therapy is its ability to achieve therapeutic nutrient concentrations that are unattainable through oral administration. High-dose vitamin C therapy delivered intravenously can achieve plasma levels up to 100 times higher than those achieved orally, offering valuable therapeutic benefits in immune enhancement, accelerated wound healing, and as an adjunctive treatment in cancer management [22]. Clinical studies indicate that IV administration of vitamin C at doses between 25-50 grams can substantially enhance immune function, reduce inflammation, and reinforce the body's antioxidant defenses [22,23]. Additionally, IV administration effectively avoids GI side effects, such as osmotic diarrhea, which commonly limit high-dose oral vitamin C supplementation [22]. IV therapy also enables the administration of other nutrients, such as glutathione, a powerful antioxidant frequently administered alongside vitamin C, providing enhanced detoxification support and reducing oxidative stress [24].

IV vitamin therapy proves highly effective in treating nutrient deficiencies, particularly in populations at increased risk due to malabsorption disorders, post-bariatric surgery, or chronic health conditions [9]. Patients with IBD, celiac disease, gastroparesis, or altered gut anatomy following bariatric surgery frequently face challenges in maintaining optimal nutrient status through oral supplementation alone [18]. IV therapy provides a reliable solution for rapidly correcting such deficiencies, notably for water-soluble vitamins like B12, folate, and vitamin C. Neurological complications linked to vitamin B12 deficiency, such as cognitive impairment and peripheral neuropathy commonly observed in conditions like pernicious anemia, can also be swiftly and effectively corrected through IV administration [25].

Beyond correcting deficiencies, IV vitamin therapy is linked to enhanced clinical outcomes across a range of health and wellness applications. IV administration of vitamin C, for example, significantly enhances leukocyte function, bolstering the immune system's capacity to combat infections and inflammatory processes [26,27]. Similarly, IV supplementation of amino acids, B vitamins, and magnesium is beneficial for athletes and individuals engaged in strenuous physical activities, facilitating muscle recovery and boosting energy metabolism [16,27]. Additionally, IV glutathione and vitamin C administration can significantly support skin health by promoting collagen synthesis, reducing oxidative damage, and offering anti-aging benefits [24].

Finally, a significant strength of IV vitamin therapy lies in its customizability, allowing healthcare providers to formulate individualized nutrient combinations tailored to specific patient needs. Customized formulations, such as the widely recognized Myers’ Cocktail, integrate multiple vitamins and minerals to target diverse conditions, including chronic fatigue, migraine headaches, and chronic stress, thereby delivering synergistic therapeutic effects uniquely suited to each patient's clinical profile and wellness goals [28-30].

Disadvantages, safety considerations, and potential risks of IV vitamin therapy

While IV vitamin therapy holds recognized clinical value for medical purposes such as treating dehydration, correcting nutrient deficiencies, and administering certain medications, its growing popularity in non-medical contexts, such as wellness centers and spas, raises several significant concerns regarding its disadvantages, safety considerations, and potential health risks.

Disadvantages

One key disadvantage of IV vitamin therapy in wellness contexts is the lack of solid evidence supporting its benefits for healthy individuals. Despite claims that IV therapy can enhance energy, boost immunity, or improve skin health, these purported benefits are primarily anecdotal or based on self-reported outcomes rather than well-designed randomized clinical trials [7]. Thus, there remains insufficient scientific support for the long-term efficacy or necessity of IV nutrient therapy for general wellness in individuals who are otherwise healthy.

Moreover, IV vitamin therapy is typically expensive, with individual sessions often costing between $100 to over $300, depending on the formulation and provider [8,31]. Since such therapies are rarely covered by health insurance, their cost-effectiveness compared to oral supplementation or improvements in diet and lifestyle habits is questionable. Additionally, the invasive nature of IV therapy, necessitating needle insertion, can cause discomfort and requires a controlled, sterile environment alongside professionally trained medical personnel, further complicating convenience and accessibility [32]. The reported beneficial effects, such as temporary increases in energy or hydration, are usually transient and should not be considered replacements for fundamental sustainable health practices, including balanced nutrition, regular physical activity, and sufficient rest [7].

Safety Considerations and Potential Risks

The safety of IV vitamin therapy is another area of concern. Proper administration requires rigorous adherence to sterility protocols to minimize complications, such as infections or abscess formation [33]. In wellness-focused settings like spas or mobile home services, adherence to sterile techniques might fall short of medical standards, potentially increasing risks. Additionally, IV therapy must always be performed by qualified, licensed healthcare professionals, as improper insertion techniques, lack of medical oversight, or errors in dosing can lead to complications including thrombophlebitis, air embolisms, or other serious medical events [32]. Equally important, IV nutrient therapy should ideally be medically indicated, as using such therapies without clear clinical justification can expose individuals to unnecessary risks and may obscure underlying medical conditions that require proper diagnosis and treatment [32].

Potential risks associated with IV vitamin therapy must also be seriously considered. Infection remains one of the most critical risks, particularly when strict sterile techniques are not adhered to, potentially leading to severe conditions such as bloodstream infections or sepsis [33,34]. Furthermore, repeated IV therapy sessions can result in vein-related complications such as phlebitis (inflammation), bruising, and even vein damage or collapse, especially if the procedure is performed incorrectly or too frequently [33]. Additionally, excessive administration of nutrients, particularly fat-soluble vitamins like A, D, E, and K, can accumulate in tissues and lead to toxic effects. Similarly, improper fluid infusion can cause electrolyte imbalances or fluid overload, negatively impacting cardiovascular and renal functions [35]. There is also a potential for allergic reactions to components within IV formulations, ranging from mild symptoms to severe anaphylaxis [34]. Lastly, individuals might develop a false sense of security from IV treatments, mistakenly viewing them as quick fixes and consequently neglecting essential aspects of health maintenance such as proper nutrition, regular exercise, and adequate sleep.

Given these considerations, careful assessment by qualified healthcare providers is essential before initiating IV vitamin therapy, emphasizing medical necessity and ensuring patient safety and informed consent.

Future prospects of IV nutrient therapy

While IV vitamin therapy has demonstrated promising benefits in certain patient populations, particularly those with clinical needs such as malabsorption syndromes, severe dehydration, or nutrient deficiencies, the future of IV vitamin therapy as a tool for general wellness and preventive health remains uncertain. There is growing public interest in the use of IV infusions to enhance energy, cognition, immunity, and skin health, but current scientific evidence to support these claims in healthy individuals is limited. To fully understand its potential, future research must address several key areas. First, robust long-term clinical trials are essential to evaluate both the safety and efficacy of IV vitamin therapy in non-hospital settings. While short-term improvements in subjective well-being have been reported, it is unclear whether these effects persist over time or if repeated use could result in adverse outcomes, such as nutrient overload or vein damage. Secondly, there is a critical need for studies that establish optimal dosing strategies. The amounts and combinations of vitamins and minerals used in IV cocktails vary widely between providers, often without standardized guidelines or individualized assessments. Determining appropriate dosages for different population groups-based on age, health status, and existing nutrient levels-would help minimize risks and ensure effectiveness. Moreover, comparative effectiveness studies are needed to assess how IV vitamin therapy performs relative to oral supplementation or dietary modifications. Such trials would clarify whether the higher cost and invasive nature of IV vitamin therapy offer any significant advantage over traditional approaches in delivering nutrients to the body. Understanding the bioavailability differences between oral and IV routes, especially in healthy individuals versus those with digestive impairments, would provide crucial clinical insight. In addition, research into the placebo effect and psychological components of IV vitamin therapy is warranted. The perceived “instant boost” that many users report may be partly influenced by expectation, environment, or the premium service model offered by IV lounges. Studies that use double-blind, placebo-controlled methodologies could help distinguish real physiological outcomes from subjective experiences. Beyond individual health outcomes, public health research should also consider the economic and ethical implications of expanding IV vitamin therapy into wellness markets. With the rise of mobile IV services and walk-in clinics offering elective infusions, questions about regulation, provider training, and access equity must be addressed. Future research should explore whether widespread use of IV vitamin therapy in the absence of medical need diverts attention from evidence-based health interventions, such as nutrition education and preventive care. Lastly, technological advancements may influence the future of IV vitamin therapy by enabling better monitoring of nutrient levels, tailoring treatments through AI-based platforms, or integrating wearable devices to optimize timing and dosing. As the wellness industry continues to commercialize IV vitamin therapy, it is vital that scientific inquiry keeps pace, ensuring that public enthusiasm is matched by rigorous, evidence-based guidance. Overall, while IV vitamin therapy holds promise in specific contexts, its expansion into the general population demands careful, comprehensive research to confirm its benefits, define best practices, and ensure its safe and effective use in both clinical and consumer settings.

## Conclusions

IV vitamin therapy shows promising clinical benefits in addressing specific medical needs such as nutrient deficiencies, malabsorption syndromes, and severe dehydration. However, its effectiveness and safety as a general wellness tool for healthy individuals remain uncertain, with current scientific evidence limited and largely anecdotal. To clarify these uncertainties, rigorous long-term clinical trials are necessary to evaluate both safety and sustained efficacy. Future studies should determine optimal dosages tailored to individual needs, clearly compare IV therapy with oral supplementation and dietary approaches and distinguish genuine physiological benefits from potential placebo effects. Additionally, expanding IV vitamin therapy into wellness markets warrants critical assessment of its economic, ethical, and regulatory implications. Ultimately, comprehensive, evidence-based research is essential to guide its safe, appropriate, and effective use.

## References

[1] Tardy AL, Pouteau E, Marquez D, Yilmaz C, Scholey A (2020). Vitamins and minerals for energy, fatigue and cognition: a narrative review of the biochemical and clinical evidence. Nutrients.

[2] Lukaski HC (2004). Vitamin and mineral status: effects on physical performance. Nutrition.

[3] Said HM (2011). Intestinal absorption of water-soluble vitamins in health and disease. Biochem J.

[4] Masri OA, Chalhoub JM, Sharara AI (2015). Role of vitamins in gastrointestinal diseases. World J Gastroenterol.

[5] Craig WJ (2010). Nutrition concerns and health effects of vegetarian diets. Nutr Clin Pract.

[6] Prescott JD, Drake VJ, Stevens JF (2018). Medications and micronutrients: identifying clinically relevant interactions and addressing nutritional needs. J Pharm Technol.

[7] Karasiewicz M, Lipiak A, Jóźwiak P, Giernaś B, Cofta M, Chawłowska E (2024). Health professionals' perspectives on commercially available intravenous nutrient therapies: a preliminary report. Healthcare (Basel).

[8] Sharma A, Marshall TS, Khan SS, Johns B (2014). Cost effectiveness of paricalcitol versus cinacalcet with low-dose vitamin D for management of secondary hyperparathyroidism in haemodialysis patients in the USA. Clin Drug Investig.

[9] Toledo-Pereyra LH (2008). Exercitatio Anatomica De Motus Cordis et Sanguinis in Animalibus surgical revolution. J Invest Surg.

[10] Daly WJ (2008). The Black Cholera comes to the Central Valley of America in the 19th century - 1832, 1849, and later. Trans Am Clin Climatol Assoc.

[11] Janakan G, Ellis H (2013). Dr Thomas Aitchison Latta (c1796-1833): pioneer of intravenous fluid replacement in the treatment of cholera. J Med Biogr.

[12] Millam D (1996). The history of intravenous therapy. J Intraven Nurs.

[13] Foëx BA (2000). Discovery of the intraosseous route for fluid administration. J Accid Emerg Med.

[14] Krajmalnik-Brown R, Ilhan ZE, Kang DW, DiBaise JK (2012). Effects of gut microbes on nutrient absorption and energy regulation. Nutr Clin Pract.

[15] Padayatty SJ, Sun H, Wang Y (2004). Vitamin C pharmacokinetics: implications for oral and intravenous use. Ann Intern Med.

[16] Rosanoff A, Dai Q, Shapses SA (2016). Essential nutrient interactions: does low or suboptimal magnesium status interact with vitamin D and/or calcium status?. Adv Nutr.

[17] Hadadi N, Berweiler V, Wang H, Trajkovski M (2021). Intestinal microbiota as a route for micronutrient bioavailability. Curr Opin Endocr Metab Res.

[18] Brown K, DeCoffe D, Molcan E, Gibson DL (2012). Diet-induced dysbiosis of the intestinal microbiota and the effects on immunity and disease. Nutrients.

[19] Fouhy LE, Mangano KM, Zhang X, Hughes BD, Tucker KL, Noel SE (2023). Association between a calcium-to-magnesium ratio and osteoporosis among Puerto Rican adults. J Nutr.

[20] Schijns W, Boerboom A, de Bruyn Kops M (2020). A randomized controlled trial comparing oral and intravenous iron supplementation after Roux-en-Y gastric bypass surgery. Clin Nutr.

[21] Wintergerst ES, Maggini S, Hornig DH (2006). Immune-enhancing role of vitamin C and zinc and effect on clinical conditions. Ann Nutr Metab.

[22] Duconge J, Miranda-Massari JR, Gonzalez MJ, Jackson JA, Warnock W, Riordan NH (2008). Pharmacokinetics of vitamin C: insights into the oral and intravenous administration of ascorbate. P R Health Sci J.

[23] González MJ, Berdiel MJ, Miranda-Massari JR, López D, Rodríguez-López JL, Adrover-López PA, Duconge J (2016). High dose intravenous vitamin c treatment in a patient with lung cancer: a case report. Clin Case Rep Rev.

[24] Wu G, Fang YZ, Yang S, Lupton JR, Turner ND (2004). Glutathione metabolism and its implications for health. J Nutr.

[25] Moore E, Mander A, Ames D, Carne R, Sanders K, Watters D (2012). Cognitive impairment and vitamin B12: a review. Int Psychogeriatr.

[26] Carr AC, Maggini S (2017). Vitamin C and immune function. Nutrients.

[27] Elhassan YS, Kluckova K, Fletcher RS (2019). Nicotinamide riboside augments the aged human skeletal muscle NAD(+) metabolome and induces transcriptomic and anti-inflammatory signatures. Cell Rep.

[28] Suh SY, Bae WK, Ahn HY, Choi SE, Jung GC, Yeom CH (2012). Intravenous vitamin C administration reduces fatigue in office workers: a double-blind randomized controlled trial. Nutr J.

[29] Padayatty SJ, Sun AY, Chen Q, Espey MG, Drisko J, Levine M (2010). Vitamin C: intravenous use by complementary and alternative medicine practitioners and adverse effects. PLoS One.

[30] Shoeibi A, Olfati N, Soltani Sabi M, Salehi M, Mali S, Akbari Oryani M (2017). Effectiveness of coenzyme Q10 in prophylactic treatment of migraine headache: an open-label, add-on, controlled trial. Acta Neurol Belg.

[31] Stollenwerk B, Iannazzo S, Akehurst R (2018). A decision-analytic model to assess the cost-effectiveness of Etelcalcetide vs. Cinacalcet. Pharmacoeconomics.

[32] Lavery I (2011). Intravenous therapy: preparation and administration of IV medicines. Br J Nurs.

[33] Lavery I (2010). Infection control in IV therapy: a review of the chain of infection. Br J Nurs.

[34] Higginson R (2011). IV therapy and infection control in patients in the community. Br J Nurs.

[35] Riegert-Johnson DL, Volcheck GW (2002). The incidence of anaphylaxis following intravenous phytonadione (vitamin K1): a 5-year retrospective review. Ann Allergy Asthma Immunol.

